# Growth and membrane fluidity of food-borne pathogen *Listeria monocytogenes* in the presence of weak acid preservatives and hydrochloric acid

**DOI:** 10.3389/fmicb.2013.00152

**Published:** 2013-06-14

**Authors:** Ioannis Diakogiannis, Anita Berberi, Eleni Siapi, Angeliki Arkoudi-Vafea, Lydia Giannopoulou, Sofia K. Mastronicolis

**Affiliations:** ^1^Food Chemistry Laboratory, Department of Chemistry, University of AthensAthens, Greece; ^2^Institute of Biology, Medicinal Chemistry and Biotechnology, National Hellenic Research FoundationAthens, Greece

**Keywords:** *Listeria monocytogenes*, membrane fluidity, phase transition, DSC, acid stress response, preservatives, weak acids, hydrochloric acid

## Abstract

This study addresses a major issue in microbial food safety, the elucidation of correlations between acid stress and changes in membrane fluidity of the pathogen *Listeria monocytogenes*. In order to assess the possible role that membrane fluidity changes play in *L. monocytogenes* tolerance to antimicrobial acids (acetic, lactic, hydrochloric acid at low pH or benzoic acid at neutral pH), the growth of the bacterium and the gel-to-liquid crystalline transition temperature point (*T*_m_) of cellular lipids of each adapted culture was measured and compared with unexposed cells. The *T*_m_ of extracted lipids was measured by differential scanning calorimetry. A trend of increasing *T*_m_ values but not of equal extent was observed upon acid tolerance for all samples and this increase is not directly proportional to each acid antibacterial action. The smallest increase in *T*_m_ value was observed in the presence of lactic acid, which presented the highest antibacterial action. In the presence of acids with high antibacterial action such as acetic, hydrochloric acid or low antibacterial action such as benzoic acid, increased *T*_m_ values were measured. The *T*_m_ changes of lipids were also correlated with our previous data about fatty acid changes to acid adaptation. The results imply that the fatty acid changes are not the sole adaptation mechanism for decreased membrane fluidity (increased *T*_m_). Therefore, this study indicates the importance of conducting an in-depth structural study on how acids commonly used in food systems affect the composition of individual cellular membrane lipid molecules.

## INTRODUCTION

*Listeria monocytogenes* has been associated with a variety of food products, including dairy foods, meat, poultry, and seafood as well as fruits and vegetables (Farber and Peterkin, 2000; Mastronicolis et al., 2011). In 2008, 1,381 confirmed human cases of listeriosis were reported in the European Union and the reported case-fatality rate was 20.5% [European Food Safety Authority (EFSA), 2010].

Modification of membrane lipid composition is clearly an important adaptation mechanism in *L. monocytogenes*, which allows it to grow in a stressful environment such as low temperature (Annous et al., 1997; Mastronicolis et al., 2005); low pH (Giotis et al., 2007; Mastronicolis et al., 2010); presence of disinfectants (Bisbiroulas et al., 2011); pressure; ion concentrations etc. (Beales, 2004). Changes in lipid composition can lead to changes in cytoplasmic membrane fluidity (Mykytczuk et al., 2007).

The term “membrane fluidity” is a convenient one to summarize a multifaceted phenomenon that has contributions from molecular packing (order) and molecular motions (viscosity; Russell, 2002). Membranes can exist in different phases and the most consistent phase transition is the one occurring when the membrane passes from a tightly ordered “gel” or “solid” phase to a liquid-crystal phase which is the active state of the membrane. A widely used method for determining the phase transition temperature (*T*_m_) is calorimetry. The influence of hydrocarbon chain length, branching and unsaturation, as well as the head group of the membrane lipids on the value of *T*_m_, is considerable. In general, increasing the chain length, decreasing the branching or increasing the saturation of the chains increases the phase transition temperature (New, 1994; Mykytczuk et al., 2007).

Weak lipophilic acids can occur naturally in many fruits and vegetables and have been widely used to maintain microbial stability in low pH foods. Weak acid preservatives affect the cells’ ability to maintain pH homeostasis, disrupting substrate transport and inhibiting metabolic pathways (Beales, 2004). The effect of many weak acid preservatives is dependent on the fluidity and permeability of the cytoplasmic membrane, since it is the first barrier to encounter the stress and any sensing mechanism would be located within it (Beales, 2004; López et al., 2006). Changes in the lipid profile of the plasma membrane may alter membrane permeability and fluidity, which may in turn contribute to tolerance (Beales, 2004).

In our previous report on the effects of different acidic stresses such as hydrochloric, acetic, and lactic acid (pH 5.5) or benzoic acid (pH 7.3) on *L. monocytogenes* total, polar and neutral lipid compositional changes, our results suggest that only low pH value enhances the antimicrobial activity of an acid, though irrespective of pH, the acid adaptation response leads to a similar alteration in fatty acid composition, mainly originating from the neutral lipid class of adapted cultures (Mastronicolis et al., 2010). However, the effects of the aforementioned acidic antimicrobials on membrane fluidity in *L. monocytogenes* have not been determined and compared to date. The present work was intended to provide new data by determining and comparing modifications in *T*_m_ of *L. monocytogenes* membrane lipids (and thus alterations in membrane fluidity) in response to acid stress induced by acids such as hydrochloric, acetic, lactic, or benzoic acids and also to correlate the fatty acid compositional changes of each acid-adapted culture (from our previous data) with the lipid thermodynamic behavior in order to clarify if modifications in the membrane physical state of adapted cells act as a defense mechanism against acid stress.

## MATERIALS AND METHODS

### CULTURE OF THE ORGANISM

An avirulent strain *L. monocytogenes*, DP-L1044 (D. Portnoy, University of Pennsylvania) prepared by a transposon insertion (Camilli et al., 1991) in the parent strain (Lm10403S), was grown in brain heart infusion broth (BHI, Difco Laboratories) at 30°C (24 h). A 10 mL aliquot of this was then inoculated into 1 L of BHI broth, which was then incubated at 30°C (Lm_control_) until early stationary phase. Four aliquots (10 mL) of the same stock were then inoculated, respectively, into 1 L BHI that were adjusted to pH_initial_ 5.5 with (i) HCl (Lm_HCl_); (ii) L-lactic acid (Fluka, PA, USA; Lm_LA_); and (iii) acetic acid (Merck, PA, USA; Lm_AA_). Another 10 mL aliquot was used to inoculate 1 L BHI with the addition of 1.00 g benzoic acid (Merck, PA, USA; Lm_BA_) pH_initial_ 7.3. All the above cultures were incubated at 30°C until early stationary phase. The growth of *L. monocytogenes* for each treatment over time was determined by measuring absorbance (OD) at 600 nm.

### EXTRACTION OF TOTAL LIPIDS

From each culture, cells pelleted by centrifugation (4°C, 5877 × *g*) were washed twice in phosphate buffer (pH 7.0). Extraction of total lipids performed essentially by extraction with chloroform/methanol (2/1 v/v) and washing the extract with 0.2 volumes of water (Folch et al., 1957). After phase equilibration, the lower chloroform layer (total lipids) was dried under nitrogen.

### DIFFERENTIAL SCANNING CALORIMETRY ANALYSIS

Two sets of extracted total lipids from each acid-adapted or non-adapted culture were utilized for differential scanning calorimetry (DSC) analysis. Each set of extracted total lipids was collected from one culture, in the case of Lm_control_ and of Lm_BA_, or by harvesting two cultures in the case of Lm_AA_ and Lm_HCl_, in order to obtain the appropriate weight of lipids for DSC analysis (4–5 mg). Notably, in the case of Lm_LA_, one set of extracted total lipids was used because the appropriate weight of lipids for DSC analysis was collected by harvesting five cultures.

Portions of the samples (approximately 4 mg) were weighed in stainless-steel capsules obtained from PerkinElmer (Norwalk, CT, USA) and sealed. Thermal scans were obtained using a PerkinElmer DSC-7 calorimeter and Pyris software for Windows. All samples were scanned from -25 to 80°C until identical thermograms were obtained, using a scanning rate of 10°C min^-^^1^. The temperature scale of the calorimeter was calibrated using indium (*T*_m_ = 156.6°C) and dipalmitoylphosphatidylcholine from Avanti Polar Lipids Inc. (Alabaster, AL, USA) bilayers (*T*_m_ = 41.2°C). The following diagnostic parameters in the observed endothermic events were recorded during the phase transition and are used for the study of lipids: *T*_m_ (maximum of the temperature peak), and ΔH (the area under the peak represents the enthalpy change during the transition).

The repeatability of the thermograms and reversibility of the transitions were checked after each run by re-heating the sample after cooling. All samples were scanned a minimum of three times.

### STATISTICAL ANALYSIS

The results were evaluated by analysis of variance (ANOVA). *T*-test for unpaired observations was tested at a confidence level of 95%.

## RESULTS

**FIGURE 1:**
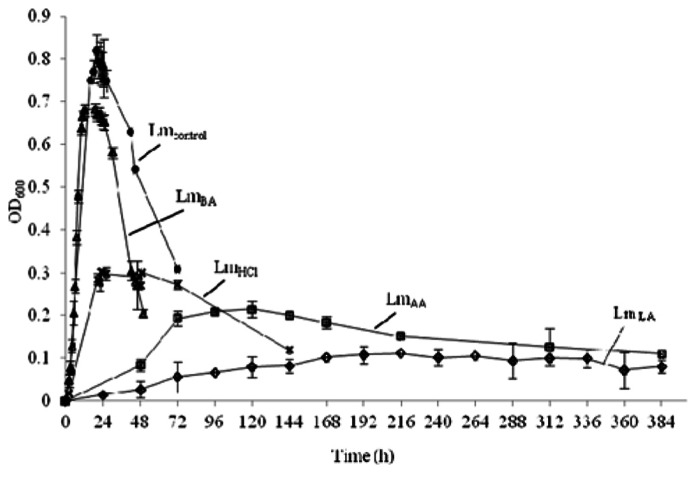
**Growth of *L. monocytogenes* before (•, Lm_control_) and after acid stress exposure by lactic (**♢, **Lm_LA_), acetic (□, Lm_AA_), hydrochloric (×, Lm_HCl_), or benzoic (**△, **Lm_BA_) acid**.

Growth of *L. monocytogenes* in BHI medium with time was determined for each treatment by measuring absorbance (OD) at 600 nm and shown in **Figure 1**. The presence of lactic, acetic, or hydrochloric acid at pH 5.5 was accompanied by low survival (*P* < 0.01), while cells grown at neutral pH in the presence of benzoic acid displayed little antilisterial activity (*P* < 0.05). The obtained OD_600_ values were at early stationary phase: Lm_control_ 0.811 ± 0.010, 10 h; Lm_LA_ 0.096 ± 0.018, 168 h; Lm_AA_ 0.217 ± 0.019, 72 h; Lm_HCl_ 0.320 ± 0.014, 24 h; and Lm_BA_ 0.694 ± 0.019, 10 h.

### Lm_control_ CELLS

**FIGURE 2:**
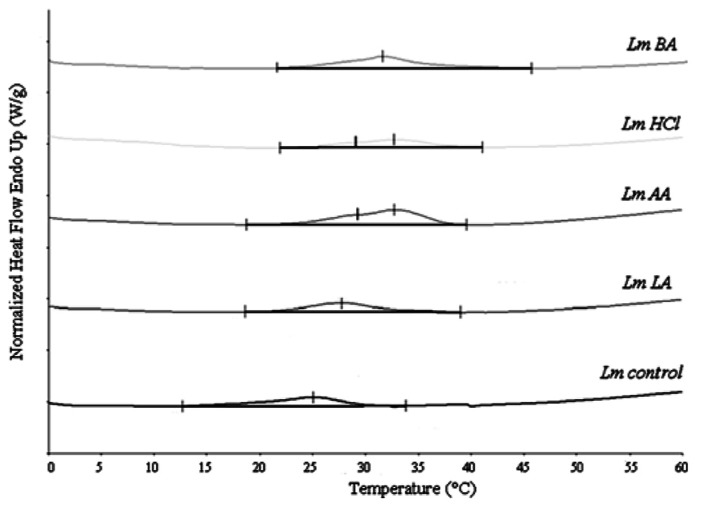
**Differential scanning calorimetry curves of *L. monocytogenes* total lipids, before (Lm_control_) and after acid stress exposure by lactic (Lm_LA_), acetic (Lm_AA_), hydrochloric (Lm_HCl_), or benzoic (Lm_BA_) acid**.

The DSC analysis revealed *T*_m_ value 25.78 ± 1.06°C as well as enthalpy difference (ΔH) 8.99 ± 0.557 J g^-^^1^ (Table 1 and **Figure 2**).

**Table 1:** Data from differential scanning calorimetry analysis of *L. monocytogenes* total lipids before (Lm_control_) and after acid stress exposure by lactic (Lm_LA_)^d^, acetic (Lm_AA_), hydrochloric (Lm_HCl_), or benzoic (Lm_BA_) acid.

	Lm_control_	Lm_AA_	Lm_HCl_	Lm_BA_
*T*_m_ (°C)	25.78 ± 1.06	29.35 ± 0.23^a^ (*T*_m1_)	29.23 ± 0.21^a^ (*T*_m1_)	30.25 ± 2.01^a^
		34.72 ± 2.28^a^ (*T*_m2_)	32.28 ± 0.56^a^ (*T*_m__2_)
ΔH (J g^-^^1^)	8.990 ± 0.557	14.921 ± 0.168^b^	8.246 ± 0.178	11.618 ± 0.401^a^
ΣBCFA/ΣSSCFA^c^	8.3	1.6	2.1	2.6

*T*_m_, *phase transition temperature; ΔH, enthalpy difference.*

a *Values statistically increased compared to* *Lm*_control_, *P* < 0.05.

b *Values statistically increased compared to Lm*_control_, *P* < 0.01.

c *Ratio of total branched-chain fatty acids, BCFA, to total saturated straight chain fatty acids, SSCFA, of total lipid fatty acid profiles of cells. These data were derived from our previous study (Mastronicolis et al., 2010)*.

d *The data for Lm*_LA_ *were*: *27.83*°*C* for *T*_m_, *7.984* J g^-^^1^ *for* ΔH, *and* 1.4 *for* Σ*BCFA/ΣSSCFA. One set of extracted lipids was utilized because the appropriate weight of lipids for DSC analysis was collected by harvesting five cultures*.

### Lm_AA_ AND Lm_HCl_ CELLS

The DSC analysis of each sample revealed two distinct peaks of increased *T*_m_ values (*T*_m1_ and *T*_m2_) compared to Lm_control_. The Lm_AA_ sample showed differences of +3.57 and +8.94°C for *T*_m1_ and *T*_m__2_, respectively (*P* < 0.05), also Lm_HCl_ sample showed differences of +3.45 and +6.50°C, respectively (*P* < 0.05).

### Lm_LA_ CELLS

In the DSC analysis an increased *T*_m_ value was measured, in which the difference was +2.05°C higher than Lm_control_.

### Lm_BA_ CELLS

In the DSC analysis an increased *T*_m_ value was measured, in which the difference was +4.47°C higher than Lm_control_ (*P* < 0.05).

As concerns the ΔH values for each instance of acid-adapted cells, the observed changes were as follows: Lm_AA_: 66% (*P* < 0.01), Lm_BA_: 29.2% (*P* < 0.05), increase compared to Lm_control_. For the rest samples the ΔH values were similar to Lm_control_ (Table 1).

## DISCUSSION

Other authors examined the antilisterial effects of these acids. Ravichandran et al. (2011) observed that benzoic acid (5 g/L) demonstrated antimicrobial activity against *L. monocytogenes* after 72 h incubation at 37°C. Heavin et al. (2009) observed that benzoic acid was more effective at inhibiting growth of *L. monocytogenes* than acetic acid, in a medium with a pH of 6.4 (acidified with HCl). Hydrochloric, lactic, and acetic acids at pH 3.5 gave similar kill curves (O’Driscoll et al., 1996). Hydrochloric acid caused low survival of *L. monocytogenes* at pH 5 (Karatzas et al., 2010) and slight antibacterial action against *L. monocytogenes* was observed with acetic acid at pH 5 (Chavant et al., 2004). In contrast, Vasseur et al. (1999) observed that the antilisterial effect was: acetic acid > lactic acid > hydrochloric acid. Similar results were observed by Bonnet and Montville (2005) in *L. monocytogenes* growing at pH 3.5. Phan-Thanh et al. (2000) also found that acetic acid had a more deleterious effect on *L. monocytogenes* than hydrochloric acid did. Exposure to lactic acid at pH 4.0 totally inactivated *L. monocytogenes*, whereas exposure at pH 4.5 had inhibitory effect (at 5 or 10°C), therefore, even small differences in pH, such as 0.5 units, may have a major impact on the survival of pathogens and hence, on food safety (Tiganitas et al., 2009). The comparative study of acid habituation of *L. monocytogenes*, under the same experimental conditions is important for the identification of differences between the survival of the pathogen, as comparison between laboratories is difficult because of variation in the assay conditions used (exact pH value, bacterial strains, incubation temperatures, etc.).

This study provides a first approach to observing the role of phase transitions of membrane lipids (membrane fluidity) in the acid adaptation response of *L. monocytogenes*. We have previously studied the lipid composition of *L. monocytogenes* cells grown in the presence of various acids (hydrochloric, acetic, lactic, and benzoic acid) and the analysis of membrane lipids revealed that *L. monocytogenes* similarly altered its fatty acid composition by incorporation more straight (mostly C_16:0_, C_18:0_, and C_14:0_) and fewer branched-chain fatty acids into its membrane independently of the acid utilized (Table 1; Mastronicolis et al., 2010). It is expected that these fatty acids changes lead to membranes with decreased fluidity and low permeability properties (Kaneda, 1991; Zhang and Rock, 2008). In the current study, the measured lipid *T*_m_ value of each set of adapted cells was increased compared to Lm_control_ and this observation is interpreted by the above fatty acid compositional changes. However, the increases in *T*_m_ values are not of equal extent and therefore are not absolutely reflected by the acyl chain compositional changes. This fact indicates that fatty acid changes may be crucial but they are not the sole mechanism by which *L. monocytogenes *perceives the acid stress (alters its membrane lipids). Furthermore, the growth of *L. monocytogenes* in the presence of hydrochloric, lactic, and acetic acid at pH 5.5 caused an increase of neutral lipid percentages (Mastronicolis et al., 2010).

Hydrochloric acid will be dissociated, whereas acetic (pK_a_ = 4.74) and lactic acid (pK_a_ = 3.79) will be undissociated at pH 5.5. The latter form of both organic acids is membrane-permeable and thus allows acetic and lactic acid to enter the microbial cell. In this work, when the cells were grown in the presence of acetic or hydrochloric acid, the highest *T*_m_ values and low survival were observed (**Figure 1**; **Table 1**), suggesting that the decrease in membrane fluidity was related to low survival. However, this tendency was reversed in the case of lactic acid, which caused the highest antimicrobial action (**Figure 1**) in *L. monocytogenes* cells and these data cannot be explained by a modification in membrane fluidity, which was minimal. This suggests that the membrane fluidity can serve only as a preliminary tool to make predictions concerning the viability of cells. Also, another interesting point was that acetic and hydrochloric acid caused two distinct phase transition points: lipids with different fatty acyls as well as different head groups, whose *T*_m_ values differ greatly from each other, undergoing phase transitions independently, and forming membranes composed of two or more separate phases. If the fatty acyls or the head groups have similar *T*_m_ values, a main transition intermediate in temperature between those of the individual components will be given (New, 1994). Mykytczuk et al. (2010) also observed decreases in membrane fluidity along with two distinct phase transition points in some strains of* Acidithiobacillus ferrooxidans* in sub-optimal pH.

Benzoic acid (pK_a_ = 4.19) at pH 7 will be in its dissociated form (benzoic anion) and this form is less membrane-permeable and thus does not facilitate its entrance to the microbial cell. The used amount of benzoic acid (1 g/L culture) did not reduce the pH of the medium. In order to reduce the pH value, even more amount of benzoic acid might be added (that is inappropriate for food systems) or one more acid should be added along to benzoic acid (that it is out of the aim of the current work, which was the study of each acid separately). Unlike the rest of the acids utilized, in the presence of benzoic acid the percentage of neutral lipid class remains constant but the decrease of negatively charged phospholipids, such as cardiolipin or phosphatidylglycerol (Mastronicolis et al., 2010), leads to a decrease in membrane fluidity, i.e., increased *T*_m_ value (New, 1994), and the data of the present study are consistent with this increase in *T*_m_. Furthermore, high *T*_m_ value and low antibacterial action (**Figure 1**; **Table 1**) was observed, suggesting that the decrease in membrane fluidity was related to the low antibacterial activity of benzoic acid. The low antibacterial action of benzoic acid might be arisen from the neutral pH of the medium. Relevant to our current work in the case of benzoic acid, Alonso-Hernando et al. (2010) also observed that decreased membrane fluidity in *L. monocytogenes* was correlated to survival upon acid stress, suggesting that adaptation to acid decontaminants is related to changes in membrane fluidity.

*Listeria monocytogenes* and *Salmonella enterica* cells exposed to sub-inhibitory concentrations of acid decontaminants (citric acid and peroxyacids) showed decreased membrane fluidity (Alonso-Hernando et al., 2010). In sub-optimal pH, a decrease in membrane fluidity of *A. ferrooxidans* was observed and this is likely linked to the overall increase in saturated fatty acids at the expense of unsaturated fatty acids (Mykytczuk et al., 2010). Adaptation to acid and starvation stress increased net cell hydrophobicity and decreased membrane fluidity of *L. innocua* (Moorman et al., 2008). ATR(+) *L. monocytogenes* cells [cells exposed to mild acid (pH 5.5), which are subsequently able to resist severe acid (pH 3.5) conditions] had lower membrane rigidities than ATR(-) cells (cells subjected at pH 3.5 directly; Najjar et al., 2009). After exposure to oregano essential oil concentrations up to 0.50%, the membrane fluidity of *L. monocytogenes* was decreased presumably to block, or at least to reduce essential oil entrance and partition into the membrane (Serio et al., 2010). Growth in the presence of butyrate, leucine, valine, isovalerate, or isobutyrate increased the calculated (theoretical estimation) transition temperature of *L. monocytogenes* cells, because of the decrease of branched-chain at the expense of saturated-chain fatty acids (Julotok et al., 2010). Increase in phase transition temperatures was observed with increased osmotic pressure in *Saccharomyces cerevisiae *(Laroche et al., 2001). Decreased membrane fluidity was also observed in *Bacillus subtilis* subjected to osmotic pressure (López et al., 2006).

An understanding of phase transitions and fluidity of membranes is important; since the phase behavior of a membrane determines such properties as permeability, fusion, aggregation, and protein binding, affects critical biochemical reactions, transport systems, all of which can markedly affect the stability of membranes, and their behavior in the cell (New, 1994; Yuk and Marshall, 2006). Acid habituation of pathogens may enhance survival in an acidic food or in the stomach and subsequently cause infection after ingestion. The resistance or adaptation of pathogens to such conditions affect food safety and thus is clearly of significance to the food industry (Beales, 2004).

Although the acid adaptation response of *L. monocytogenes* altered the fatty acid composition similarly, irrespective of the acid utilized (Mastronicolis et al., 2010), in the present study observed *T*_m_ values were increased but not equally. This suggests that the *T*_m_ value (membrane fluidity) of lipids does not depend only on the acyl constituent, but also on the total composition and nature of the lipid molecular structure (e.g., phospho-, glyco-, amino-head groups for polar lipids or the specific lipid molecule for neutral lipids, e.g., diclycerides, esters, waxes, etc.). Thus, understanding the physical chemistry of membrane lipids is important in the sense that the characteristics of lipid species, and their heterogeneity, all affect biological membranes. Our current understanding of the role of individual lipid species in a heterogeneous lipid matrix and the specific lipid–lipid and lipid–protein interactions is still far from comprehensive. Therefore, one conclusion of this study would support the in-depth identification of the membrane polar and neutral lipid molecules of *L. monocytogenes* cells in the presence of the acids utilized. Furthermore, in this study an avirulent mutant strain was used. Previous studies have revealed that this strain has similar fatty acid composition as wild strains in optimal condition of growth or in cold adaptation (Annous et al., 1997; Mastronicolis et al., 1998, 2010; Chihib et al., 2003; Julotok et al., 2010), thus we suppose that this mutation will have no impact on the present results response to acids. However, more studies may be required with more strains in order these results to be confirmed because there are not sufficient studies in this field.

In conclusion, in this study we observed that adaptive response of *L. monocytogenes* to weak or strong acid food preservatives includes an increase in the total lipid *T*_m_ (decreased membrane fluidity), decreasing the ability of the weak acid preservatives to pass through the membrane and to act into the microbial cell, and thus conferring protection. Furthermore, decreased membrane fluidity acts as strong defense mechanism in some conditions (in the cases of hydrochloric or acetic acid) or as mild defense mechanism (in the cases of benzoic or lactic acid).

## Conflict of Interest Statement

The authors declare that the research was conducted in the absence of any commercial or financial relationships that could be construed as a potential conflict of interest.
